# Superluminescence from an optically pumped molecular tunneling junction by injection of plasmon induced hot electrons

**DOI:** 10.3762/bjnano.6.111

**Published:** 2015-05-04

**Authors:** Kai Braun, Xiao Wang, Andreas M Kern, Hilmar Adler, Heiko Peisert, Thomas Chassé, Dai Zhang, Alfred J Meixner

**Affiliations:** 1 Institute of Physical and Theoretical Chemistry, University of Tübingen, Auf der Morgenstelle 18, 72076 Tübingen, Germany

**Keywords:** inelastic tunneling, light emitting diode, quantum plasmonics, scanning near-field optical microscopy, tip-enhanced Raman spectroscopy

## Abstract

Here, we demonstrate a bias-driven superluminescent point light-source based on an optically pumped molecular junction (gold substrate/self-assembled molecular monolayer/gold tip) of a scanning tunneling microscope, operating at ambient conditions and providing almost three orders of magnitude higher electron-to-photon conversion efficiency than electroluminescence induced by inelastic tunneling without optical pumping. A positive, steadily increasing bias voltage induces a step-like rise of the Stokes shifted optical signal emitted from the junction. This emission is strongly attenuated by reversing the applied bias voltage. At high bias voltage, the emission intensity depends non-linearly on the optical pump power. The enhanced emission can be modelled by rate equations taking into account hole injection from the tip (anode) into the highest occupied orbital of the closest substrate-bound molecule (lower level) and radiative recombination with an electron from above the Fermi level (upper level), hence feeding photons back by stimulated emission resonant with the gap mode. The system reflects many essential features of a superluminescent light emitting diode.

## Introduction

The emission of photons from the gap of a scanning tunneling microscope (STM) has been a focus of interest for more than twenty years [1–2] and has been used for acquiring spectroscopic information with ultra-high spatial resolution [3]. For pure metal surfaces [4–5] or organic monolayers adsorbed directly on a metal surface [6], the emission of light originates predominantly from the radiative decay of localized surface plasmons (LSP) excited by inelastic electron tunneling (IET) as the direct luminescence of the molecules is quenched. If the molecules are decoupled from the metal surface by an ultrathin dielectric spacer, intrinsic molecular luminescence can be observed down to the single-molecule level [7], showing vibronic bands [8–9]. Very recently electroluminescence from a conducting molecule, bridging the gap was observed [10]. The efficiency of photoemission is quite low, typically with a quantum efficiency (QE) per tunneling electron of the order of 10^−6^ to 10^−5^ emitted photons [2]. A different approach for ultra-high resolution optical spectroscopy has emerged recently, the so-called tip-enhanced Raman scattering (TERS) [11–12] or gap mode near-field optical microscopy [13]. This technique has attracted great interest as a means for local Raman [14–15] or luminescence spectroscopy [16] with nanometer spatial resolution. Since efficient Raman scattering from molecules in the gap requires gap widths as short as one nanometer, electron tunneling is meanwhile routinely used to control the tip–sample distance [15,17–18], or to measure in tip/specimen/substrate conduction junctions [19].

**Figure 1:**
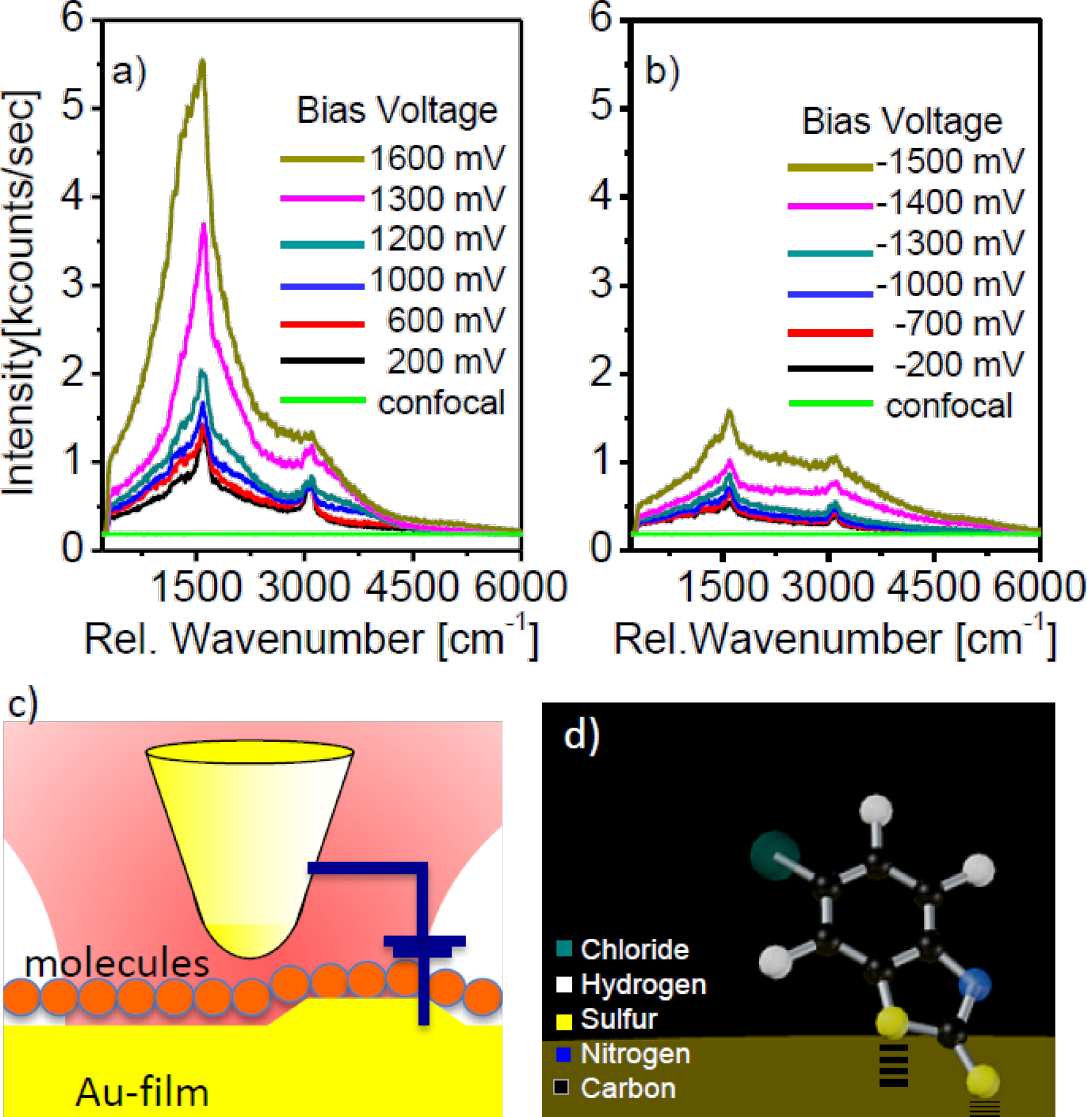
Sequences of tip-enhanced spectra (a,b) (Raman lines on luminescence background) recorded from the tunneling junction under constant laser illumination as a function of the positive and negative bias voltages, respectively. As a comparison, the confocal spectrum collected without the presence of the tip is shown as well (green lines). Typical tunneling junction (c) with a sharp gold tip statically positioned one nanometer above a gold substrate covered with a monolayer of chemisorbed Cl-MBT-molecules. Sketch of a Cl-MBT molecule (d) bound to the Au surface, after [25].

Here, we demonstrate bias-driven nonlinear optical amplification of the tip-enhanced signal from the very low number of molecules enclosed in a laser-pumped tunneling junction of a STM. For efficient excitation and collection of photons the junction was centered in the focus of a parabolic mirror used in place of the objective lens in a confocal microscope [20–23]. The experimental apparatus and conditions are described in detail in Supporting Information File 1 (Figures S1–S4). For the specimen, we have chosen a Au film covered with a monolayer of 5-chloro-2-mercaptobenzothiazole (Cl-MBT) molecules since (i) they chemically bind to the metal substrate (Figure 1c) via the sulfur atoms with the molecular plane oriented almost perpendicular to the substrate and at the same time allowing their p-electron systems to interact with the surface orbitals of the substrate [24–25]. (ii) Their first electronically excited state lies in the ultraviolet and cannot be reached by the laser photons at λ = 632.8 nm, therefore reducing photo damage and (iii) they have a static electric dipole moment with a negative partial charge located on the Cl atom bound to the benzene ring.

## Results and Discussion

**Figure 2:**
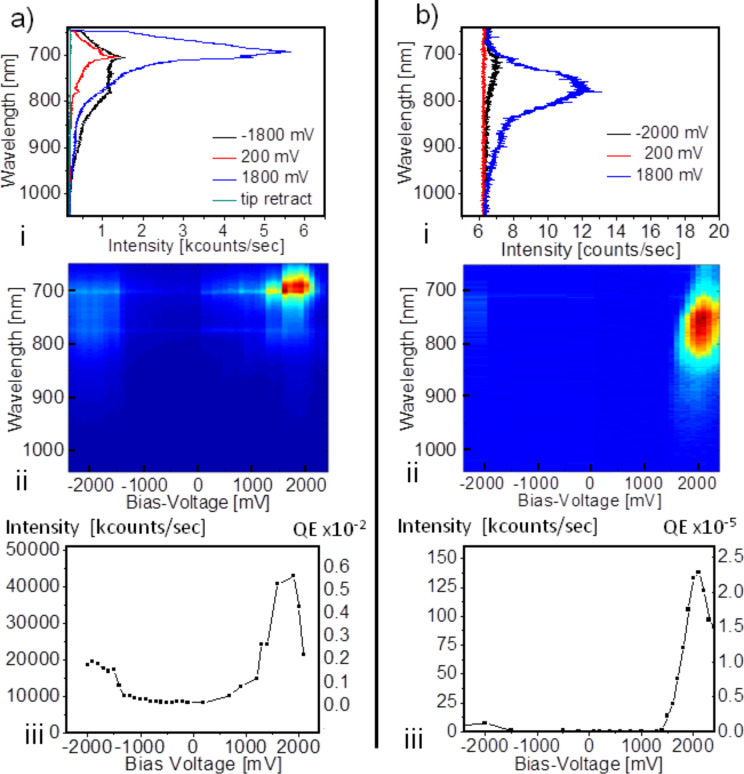
TERS spectra (a) from the tunneling junction excited at λ = 632.8 nm as a function of the bias voltage (i), (ii) with the respective spectrally integrated intensity trajectory (iii). Electroluminescence spectra excited by inelastic tunneling (b) without laser illumination as a function of the bias voltage (i), (ii). All spectra were recorded with the same tunneling current (1 nA) and are normalized to 1 s integration time. The corresponding *QE* are defined as the number of Stokes-shifted photons per tunneling electron and can directly be compared for the two situations. Note the very different intensity scales.

When a positive bias voltage is applied at the tunneling junction and steadily increased while the tunneling current of 1 nA is kept constant, the negatively charged part of the molecules experiences an increasing attractive force towards the positively charged tip. In the low bias-voltage range, i.e., for |*U*_b_| < 1000 mV, the spectra (Figure 1a and Figure 1b) change only moderately irrespective of the polarity of the applied voltage and one can observe the typical tip-enhanced Raman bands of Cl-MBT residing on a broad luminescence background. When *U*_b_ exceeds +1200 mV we observe a strong intensity increase of more than one order of magnitude affecting mainly the aromatic Raman bands around 1600 cm^−1^ and hence leading to narrowing of the total spectrum (Raman plus luminescence). In contrast for negative bias mainly the luminescence background increases leading to a spectral broadening (Figure 1b, Figure 2a). Certainly, the increasing bias voltage is slightly increasing the distance between the tip and the surface, but this displacement is only about 0.1 nanometer (see Figure S3, Supporting Information File 1). Hence, we can rule out that the bias voltage induced increase of the emission from our junction results from a variation of the tip–sample junction. This qualitatively different spectral behavior must originate from the presence of the Cl-MBT molecule in the junction, since it is not found for the pristine Au–Au junction (see Figure S5, Supporting Information File 1).

This behavior is fully reversible up to *U*_b_ = 2000 mV. When the voltage scan is repeated with the same tunneling current but without simultaneous laser illumination, we observe a sudden onset of a broad and weak electroluminescence signal at *U*_b_ > 1500 mV due to inelastic tunneling (Figure 2b). It reaches a maximum of 138 kcounts/s at *U*_b_ = 1900 mV. Ultraviolet photoelectron spectroscopy measurements of a monolayer of Cl-MBT on a flat gold substrate indeed confirm that the energetic distribution of the highest occupied molecular orbital (HOMO) with respect to the Fermi level of the Au substrate lies between −1.2 and −2.0 eV and the d-bands of Au lie below −2.0 eV (see Figure S6, Supporting Information File 1). These values are consistent with the intensity maximum in panel (iii) of Figure 2b. In agreement with theoretical predictions [26–27] the sharp onset of the electroluminescence signal marks the potential difference that has to be overcome by the anode for hole injection into the HOMO of the molecule located at the narrowest position of the tip–sample gap and for which an effective current starts to flow through this molecule. At a bias voltage of *U*_b_ = 1800 mV electrons near the Fermi energy undergo radiative recombination with the hole, which gives rise to the intensity maximum at around 775 nm observed in the blue spectrum in Figure 2b. If the bias voltage is inverted, (negative tip and positive sample) we observe only a very weak electroluminescence band centered at 725 nm. In contrast, electroluminescence from a pure Au/Au junction induced by inelastic electron tunnelling is independent of the tip polarity [1–5]. The situation here is analogue to a light emitting diode (LED) consisting of a pn-junction. When a voltage is applied to the anode lead of the LED that is more positive than the voltage applied to the cathode lead by at least the forward voltage drop of the LED, a current flows and results in emitted photons with an energy equivalent to the band gap. The quantum efficiency of 10^−5^ shown in panel (iii) of Figure 2b is comparable to the state of the art [2,10].

Comparing the data in Figure 2a and Figure 2b we note a dramatic effect of the incident radiation: For a positive bias the voltage-dependent intensity increase of the laser-induced spectrum (i.e., the TERS-spectrum and its luminescence background) is about three orders of magnitude larger than the electroluminescence signal at *U*_b_ = 1800 mV under identical tunneling current, which is equivalent to a *QE* of almost 10^−2^ photons per tunneling electron. Furthermore, for increasing positive bias voltages the luminescence background concentrates at the spectral maximum of the strongest Raman bands giving a peak maximum at 700 nm (Figure 2a, panel (ii)). It is distinctively different to the electroluminescence band obtained at *U*_b_ = 1800 mV that has a red-shifted maximum at 775 nm and a three times larger half-width. Both effects cannot be explained simply adding the TERS signal and its background at low bias voltage and the electroluminescence. Thus, an additional mechanism must be involved in the bias-voltage-dependent increase of the laser-induced emission. Recently, it has been shown that plasmon-excited nanoparticles can be an efficient source of hot electrons [28–29]. Consequently, illuminating the tip/sample junction with a focused laser beam polarized along the axis of the tip, a coupled surface plasmon oscillation in the Au tip and the underlying sample Au surface is induced. It manifests itself as a highly localized surface charge oscillation at the very apex of the tip and the Au surface below and constitutes a laser-power dependent source of hot electrons that can recombine more efficiently with the hole in the junction molecule than the conduction electrons.

**Figure 3:**
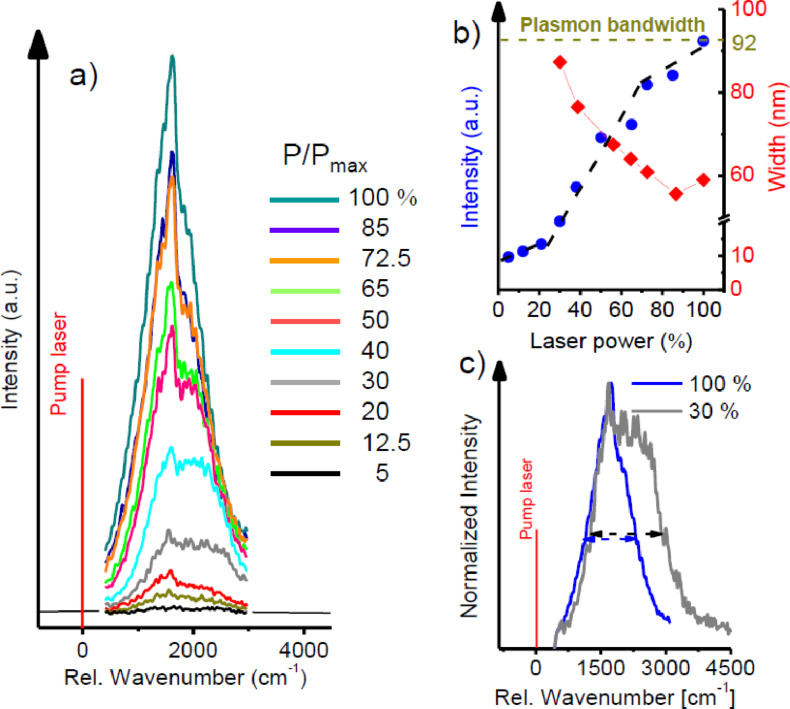
TERS spectra (a) recorded as a function of optical pump power (in % of the maximum pump power of 300 μW) for a constant bias voltage of +1800 mV. Spectrally integrated intensity (blue dots) and the full width at half maxium (FWHM) (red diamonds) of the of the luminescence peak as a function of optical pump power are given in (b). The dashed lines are guides to the eye. (c) Spectral narrowing demonstrated by overlapping the spectra recorded at 30% and at 100% of the maximum laser power.

Since the hot electrons spill over from the Au surface into the molecules [29], this situation is similar to a population inversion with a large number of electrons in an upper level (i.e., closely above the Fermi level) and a lower level of the depopulated HOMO of the molecule in the tunneling gap. As a consequence the *QE* at a bias of 1800 mV is almost three orders of magnitude higher for the optically pumped junction (Figure 2). Furthermore the laser-induced signal recorded at *U*_b_ = 1800 mV and constant tunneling current shows a distinct dependence on the incident laser power (PIL) (pulse length 100 ps, 80 MHz repetition rate, maximum power 300 μW) with a nonlinear intensity increase both for the luminescence background and the Raman signal (Figure 3a). The spectrally integrated intensity of the signal Figure 3b shows a low-gain regime for an optical pump power, PIL < 25%, a high-gain regime for 25% < PIL < 75% and almost saturates for PIL > 75%. The intensity in the low-gain regime consists mainly of the luminescence from the plasmon resonance of the Au/Au-junction and the spectrum has a full width at half maximum of about 90 nm with very weak superimposed Raman peaks (Figure 3c). At higher pump powers more and more intensity of the luminescence background is focused into the spectral region of the strongest Raman bands at around 1600 cm^−1^, which is in resonance with the coupled plasmon mode of the junction at 698 nm (Figure S7, Supporting Information File 1), leading to a spectral narrowing of the luminescence of about 40% to 55 nm. Such a spectral narrowing along with the nonlinear intensity increase observed as a function of optical pump power is a reliable sign of signal amplification by positive feedback. This feedback directs a portion of the amplified photoluminescence (PL) emission back towards the metal/molecule/metal junction in resonance to the gap mode where the intensity reaches a maximum, i.e., in the spectral region of the most intense Raman lines. Indeed resonant surface plasmon amplification by stimulated emission from plasmonic nano- and microstructures has recently been predicted and experimentally realized [30–32]. Considering the fact that the Raman scattering and PL emission processes originate in the very center of the tip–substrate gap, any generated photon will firstly couple to the gap mode before being scattered to the far field. While the gap modes plasmon resonance is very broad, exhibiting a quality factor of only *Q* ≈ 15, the resonantly stored energy in the optical near field in the gap is extremely well localized, in a volume having an upper limit of approximately 4 × 4 × 1 nm^3^ (see Figure S8, Supporting Information File 1). Hence, a feedback of the emitted energy into the quantum system can occur. When our system is optically pumped, a large population of plasmon-induced hot electrons near the Fermi level is created, which can refill the hole in the HOMO. Thus, there is a feedback of energy into the gap mode by stimulated emission, which outperforms the recombination by spontaneous emission. In Figure 3 the spectral narrowing and the nonlinear intensity increase are not as pronounced as for a conventional laser with a high-*Q* resonator. Hence, the emission of our system should be called superluminescence [33] or amplified spontaneous emission [34] in perfect agreement with similar systems known from classical laser physics.

**Figure 4:**
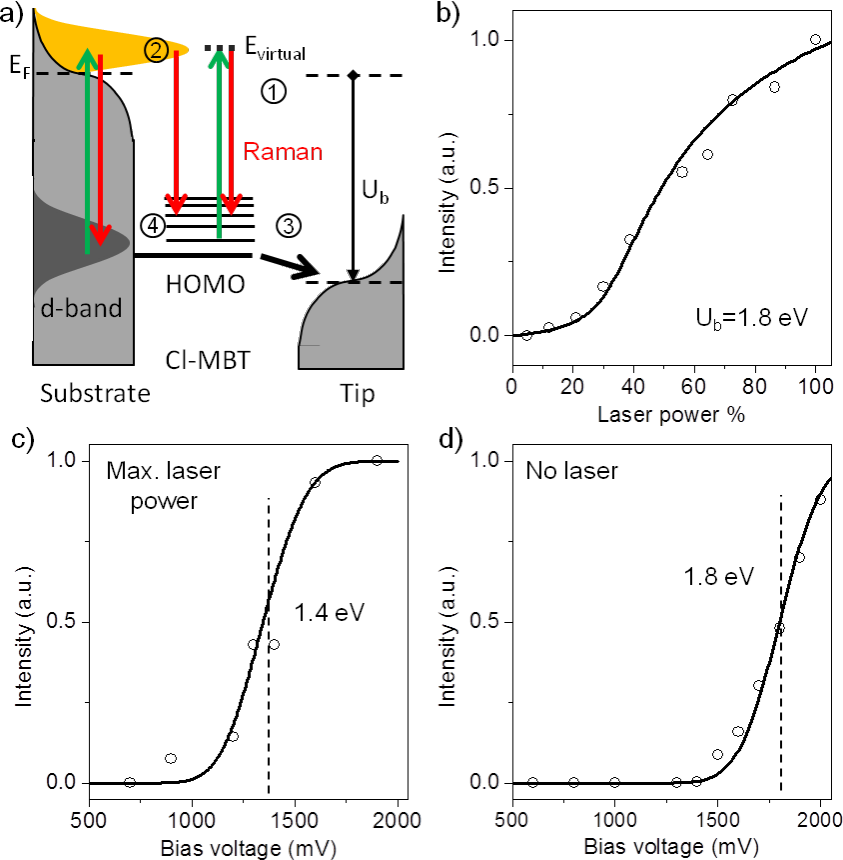
(a) Schematic energy level diagram of the gap/molecule hybrid system in a laser-illuminated tunneling junction. The normalized experimental data (open circles) and the calculated total emission (solid lines) as a function of the incident flux for fixed *U*_b_ (1.8 eV) (b), as a function of the bias voltage *U*_b_ for fixed incident flux (c) and for no laser illumination (d).

In the same way we can explain the threshold-like increase of the tip-enhanced signal as a function of the bias voltage by stimulated emission. The individual processes involved are summarized in Figure 4a. Green arrows represent processes drawing energy from the incident pump-laser field, i.e., Raman scattering from the surface-bound molecules (1) and generation of hot electrons from the d-band (2). Plasmon excitation is not shown in this figure. At low bias voltages these processes are independent and the Raman scattered photons just add to the luminescence background of the junction. However, when the Fermi level of the tip drops below the HOMO energy of a surface-bound molecule, efficient elastic electron tunneling from the molecule closest to the tip occurs (3), which leaves behind a hole in the molecule. This hole can then be refilled by a conduction electron or, more efficiently, by a hot surface electron from the Au substrate (4), emitting a photon, hence opening an additional, voltage-dependent channel for creating additional photons in the junction.

Depletion of the HOMO via elastic tunneling to the tip and generation of hot electrons by irradiating the plasmonic system with incident laser light apparently leads to a population inversion between an empty HOMO-level and the higher energy level of hot electrons; thus gain is expected under the very conditions under which enhanced emission can be observed experimentally: TERS at 1600 cm^−1^ from neighboring molecules spectrally overlap with the resonance of the junction and acts as an optical seed signal in the gap mode.

This leads to an amplification of the most intense spectral component and the spectral narrowing of the total amplified signal (Figure 2a and Figure 3) in the metal/molecule/metal junction for a positive bias voltage above threshold. For a negative bias voltage, electrons must tunnel directly from the tip to the metal substrate, leading to the broad luminescence in Figure 1. The behavior of the molecule/gap hybrid system can be described with a coupled rate-equation model similar to a four level laser. In this approach, the population of the molecules HOMO level *P*_0_ is described by its time derivative,

[1]
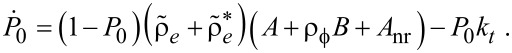


In Equation 1, the first term in brackets is the probability that the HOMO level is unoccupied. The second brackets contain the number of electrons available for recombination where 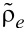 relates to the conduction-band electrons and is independent from the incident laser power, while 
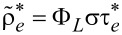
 relates to the number of hot electrons in the tunneling junction depending on the photon flux through the focus Φ_L_, the effective absorption coefficient, σ, taking into account the near-field effect and the lifetimes of the hot electrons, 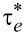. From the incident radiation and the spectrally integrated emission intensity of the pure junction, σ is estimated to be on the order of 10^−6^. In our case, the hot electrons are created highly localized close to the surface and have energies just above the Fermi level. Hence, their life time, 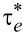, due to radiative and nonradiative decay is on the order of 1 ps which is much longer compared to the bulk [35–36]. The third brackets in Equation 1 contain the Einstein coefficients for spontaneous recombination, *A*, and stimulated recombination, *B*, as well as the corresponding coefficient for spontaneous nonradiative recombination, *A*_nr_, which are related to, 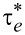, and the photonic energy density, 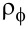, in the gap. The last term describes the HOMO levels depletion through tunneling to the tip. The tunneling rate constant can be expressed as:

[2]
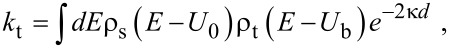


where 
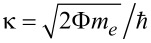
, *E* is the energy, ρ_s_ is the density of electrons in the tunnelling source (i.e., the HOMO of the molecule), *U*_0_ is the energy difference between Fermi and HOMO levels, ρ_t_ is the density of empty states in the tunneling target (i.e., the density of holes in the tip), *d* is the width of the barrier, Φ its height and *m_e_* is the electron mass. Hence, *k*_t_ can be consequently written by the HOMO states of the molecule represented here by a Gaussian function and the bias voltage *U*_b_ in the form of a Fermi–Dirac distribution as:

[3]
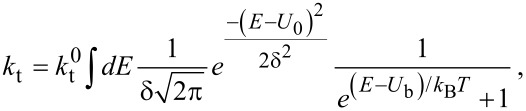


where 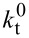 is a constant as ln A / e = 6.24 × 10^9^ s^−1^, 
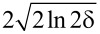
 is the width of the HOMO, *k*_B_ is the Boltzmann constant and *T* is the temperature as 300 K. The energy density of the optical field in the gap


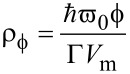


is a dynamic system itself, described by the relation:

[4]
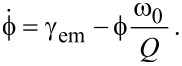


Here, ω_0_ is the resonance frequency (at 698 nm, see Figure S7, Supporting Information File 1) of the junction, Γ = (ω_0_/Q) its spectral bandwidth, *Q* its quality factor and *V*_m_ its spatial volume. The total emission rate γ_em_ comprises all optical emission processes in the gap, i.e., spontaneous and stimulated recombination as well as Raman scattering (Φ_L_σ_R_). From the measured Raman intensity, the effective Raman scattering coefficient in the tunneling junction taking the near field enhancement into account is estimated as σ_R_ ≈ 10^−9^.

Having found the steady-state solution to Equation 1 and Equation 3, the total radiative yield γ_em_ of the hybrid system at different bias voltages and incident flux can be quantitatively determined. Without laser illumination, the total emission is proportional to the tunneling rate *k*_t_ from the HOMO state of the molecule to the tip. With the fitting parameters *A* = 2.5 × 10^11^ s^−1^, *B* = 4.95 × 10^24^ m^2^/J·s^2^, *A*_nr_ = 10^12^ s^−1^, δ = 0.15 eV, *U*_0_ = 1.4 eV (with laser), *U*_0_ = 1.8 eV (without laser), this model reproduces the nonlinear increase of the Stokes shifted signal for both situations as a function of the incident laser power (Figure 4b) and the bias voltage (Figure 4c and Figure 4d). The smaller fitting value of *U*_0_ for the junction with laser illumination is consistent with the hot electron population above the Fermi level requiring less bias voltage. The system reflects many essential features of a superluminescent light emitting diode and is an important step towards an efficient and fast optical point-light source.

## Conclusion

In summary, our results demonstrate how optical enhancement inside the plasmonic cavity can be further increased by localization via tunneling through a molecule. We anticipate that stimulated emission from a tunneling junction will advance our fundamental understanding of quantum plasmonics and lead to new analytical applications. Furthermore, this concept represents the basis for novel ultra-small, fast, optically and electronically switchable devices which could find applications in high-speed signal processing and optical telecommunications.

## Supporting Information

File 1 Additional experimental data.
