# Supplementary material for: Superluminescence from an optically pumped molecular tunneling junction by injection of plasmon induced hot electrons
Source: Beilstein J Nanotechnol. 2015 May 4;6:1100–6. doi: 10.3762/bjnano.6.111 (PMC4463973; doi:10.3762/bjnano.6.111)
Supplement: File 1 — Additional experimental data. [file Beilstein_J_Nanotechnol-06-1100-s001.pdf]

Supporting Information

for

Superluminescence from an optically pumped molecular tunneling junction by injection of plasmon induced hot electrons

Kai Braun, Xiao Wang, Andreas M. Kern, Hilmar Adler, Heiko Peisert, Thomas Chassé, Dai Zhang, and Alfred J. Meixner*

Address: Institute of Physical and Theoretical Chemistry, University of Tübingen, Auf der Morgenstelle 18, 72076 Tübingen, Germany

Email: Alfred Meixner* - Alfred.Meixner@uni-tuebingen.de

* Corresponding author

Additional experimental data

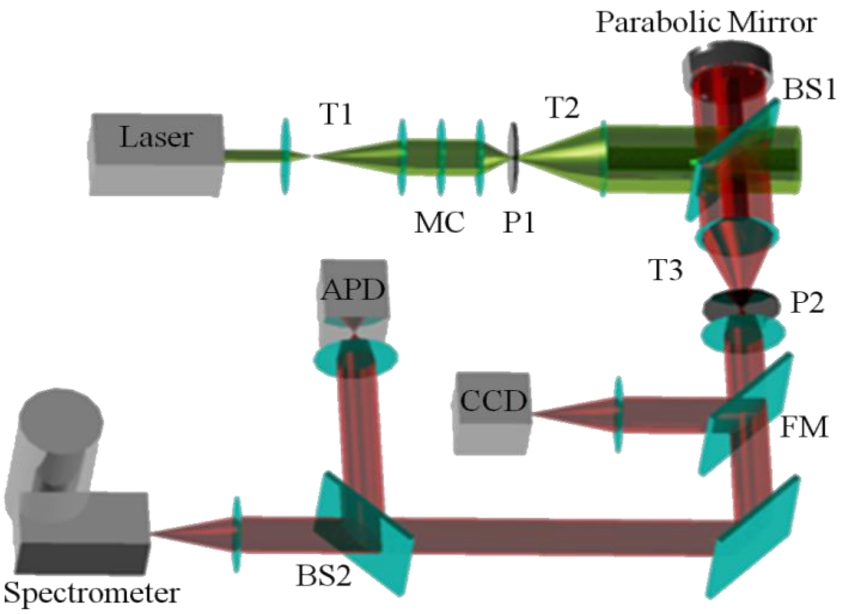

Figure S1: All measurements were performed with a custom-built parabolic mirror scanning near-field optical microscope equipped with an also homebuilt STM scanner. Two laser sources were used: a) helium-neon laser (Melles Griot 25LHP991-230) at 632.8 nm (300 µW max. on the sample) as a cw excitation source and b) a pulsed diode laser (Picoquant LDH-D-C-640) at 635 nm (controllable output power up to 300 µW on the sample). Integration time per individual spectrum was between 1 s and 3 s. All spectra were acquired by an energy calibrated spectrometer (Acton SpectralPro SP2300) with an attached intensity calibrated CCD camera (Princeton Instruments Spec-10).

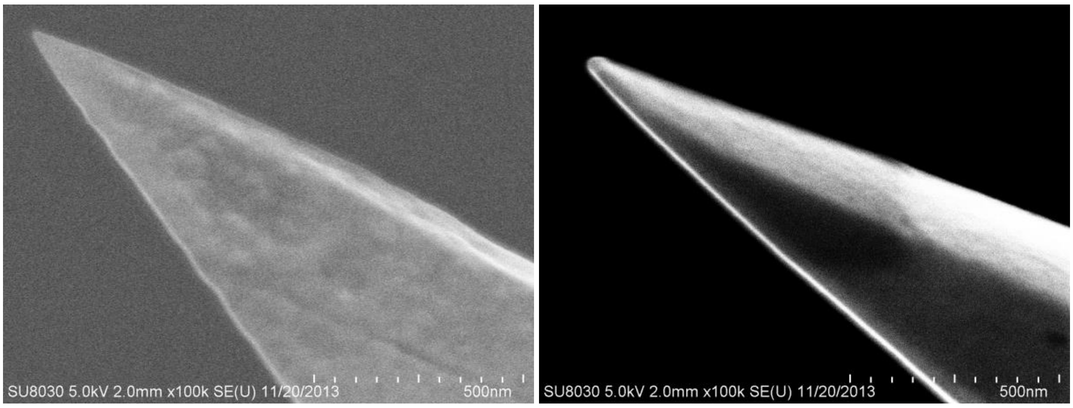

Figure S2: The gold tips were prepared by electrochemical etching in a fuming HCl solution (Sigma Aldrich Art.-Nr. 84415). All used tips are characterized by an SEM (Hitachi SU8030) to check their roughness and geometry.

For recording a sequence of spectra the tip was positioned statically above the sample surface at a fixed position and the tunnelling current was kept constant at 1 nA. A 200 nm thick gold layer was evaporated on a silicon wafer as the substrate. These substrates were then dipped for 10 min in a 10^{-2} M solution of Cl-MBT (Sigma Aldrich, 90% technical grade) molecules in Uvasol methanol. Afterwards, the samples were carefully rinsed with methanol and dried in a clean-air box. With this proven procedure we produced self assembled molecular monolayer’s where the Cl-MBT molecules bind via their sulphur atoms to Au substrate and the molecular plane oriented away from the surface plane [1].

Tip–sample separation

The tunnelling rate increases with the applied voltage and decays exponentially with the gap distance [2]. For a bare Au-Au-junction, we find for a constant current of 1 nA and a bias voltage of |200 mV| a tip-sample distance of one nanometer. Hence, since we keep the tunnelling current fixed at 1 nA the tip sample distance increases (see Figure S3) with the bias voltage by ca. 0.1 nm for |2000mV|. This would only slightly decrease the coupling strength and hence the enhancement of the optical signal from

the junction, which is even the opposite of what we observe. Hence we can rule out that the bias voltage induced increase of the emission from our junction results from a variation of the tip-sample junction.

$$I \propto U_T \rho(0, E_F) e^{-2kd} \text{ mit } k \approx \frac{\sqrt{2m\Phi}}{\hbar} \qquad \text{(F1)}$$

I = current, UT = bias voltage, ρ(0,EF) = local density of states (konstant), d = distance between tip and surface, m = electron mass,Φ = work function (gold 5.1 eV)

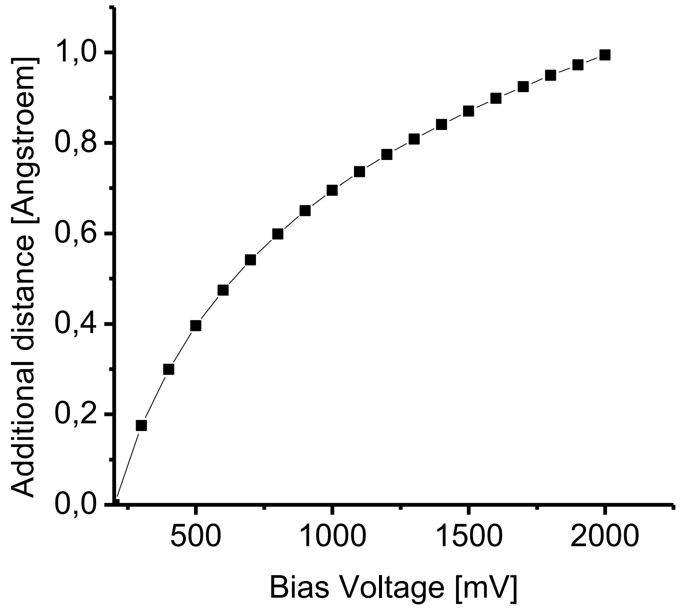

Figure S3: Calculated increased tip–sample distance with increasing bias voltage according to (F1) taken from [2]

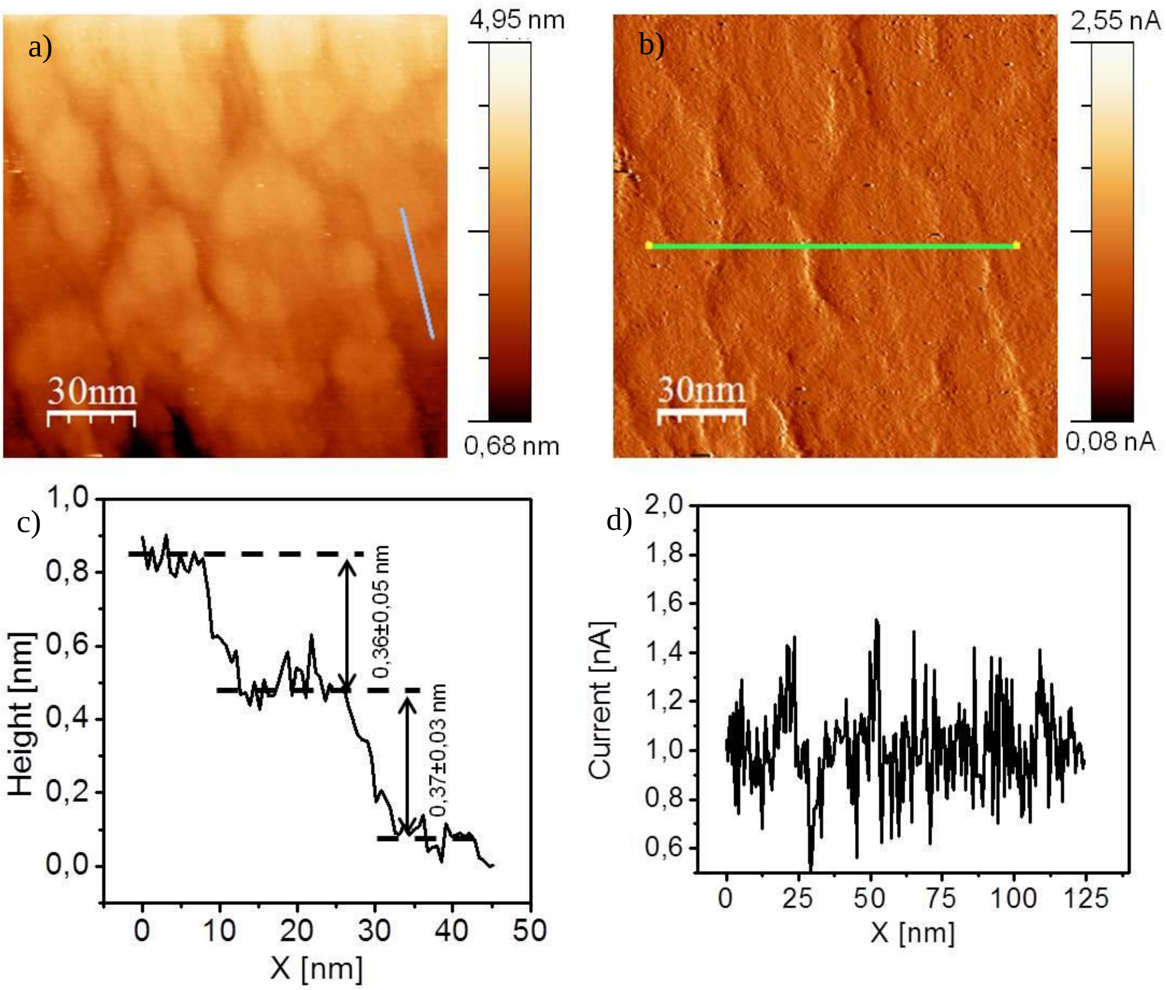

Figure S4: STM measurement on an evaporated Au film functionalized with SAM of Cl-MBT-molecules. The topography images (a) shows in the marked line-section clearly reproducible regular steps (c) and reasonable sized flat terraces were the experiments take place. The current image (b) and the corresponding linesection (d) demonstrate a stable constant current on the SAM.

Comparison with a molecule free Au/Au-junction

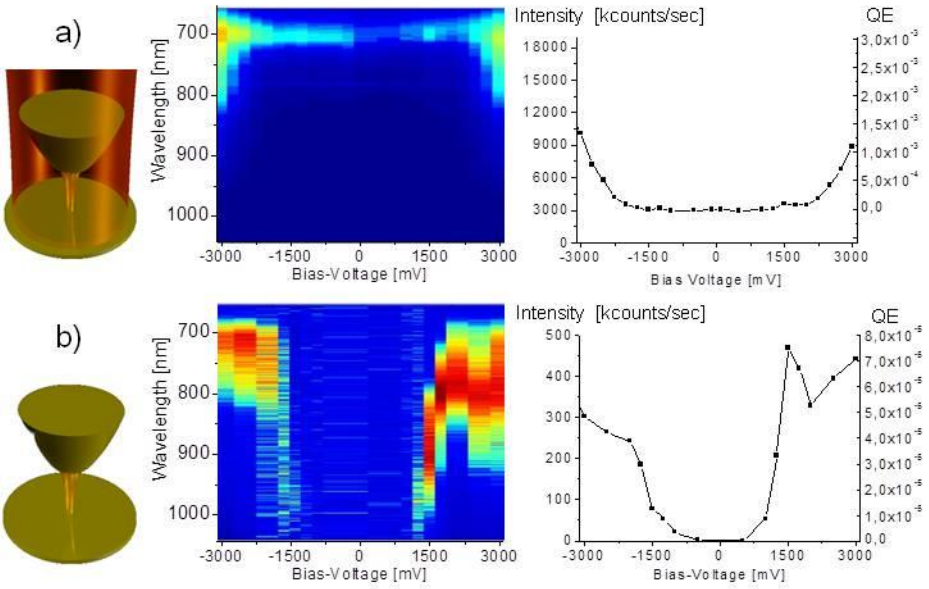

Figure S5: (a) Tip-enhanced photoluminescence spectra for a molecule free Au-sample/Au-tip junction and (b) electroluminescence spectra excited by inelastic tunneling without laser illumination as a function of the bias voltage. All spectra were recorded with a tunneling current of 1 nA and are normalized to 1s integration time. In panel (a) the laser power at the sample was 270 μW. Both spectral series are clearly non polarity dependent as opposed to the spectral series recorded from the Au / SAM / Au junction shown in Figure 1 & 2 of the main text. The electroluminescence spectra of the pure Au junction show typical spectral fluctuations described in the literature [3,4].

Quantum efficiency [QE] per tunnelling electron

In the following we calculate first the number electroluminescence photons per tunnelling electron for the pure Au-Au junction and for the Au-Cl-MBT-Au junction.

Since the luminescence intensity from the pure Au-Au junction increases by applying a bias voltage of 2500 mV, we can similarly calculate the quantum efficiency per tunnelling electron for this enhancement from the spectrally integrated number of luminescence photons at 2500 mV if we subtract the number of luminescence photons recorded at low bias. The same procedure can be used to calculate the quantum efficiency per tunnelling electron for the bias voltage dependent enhancement of the TERS-signal plus luminescence background for the Au-Cl-MBT-Au junction.

The measurements were performed under similar experimental conditions with a tunnelling current of 1 nA, and 250 μW laser power for excitation. For all experiments the same Au-tip was used. The bias voltage for the Au-Au junction was 2500 mV and for the Au-Cl-MBT-Au junction it was 1800 mV.

A tunnelling current of 1 nA corresponds to a tunnelling rate of $6.241 * 10^9 \frac{\text{electrons}}{\text{s}}$.

The optical detection efficiency of the whole system was approximately 1% (estimated by the efficiency of the detectors, spectrograph and the several mirrors and filters in the detection path). Therefore all measured intensities were multiplied by a factor of 100 to be consistent with the theoretical calculations.

Arrangement of the involved energy levels

Ultraviolet photoelectron spectroscopy (UPS) is well suitable for the determination of the energetic position of molecular orbitals at particular interfaces. UPS measurements were performed at the third-generation synchrotron radiation source BESSY II at the optics end station. Figure S6 shows the spectra of the bare gold substrate (blue) and of gold covered with about one monolayer Cl-MBT (red) taken at an excitation energy of 110 eV. The black curve is the difference of the gold spectrum and the the Cl-MBT spectrum after alignment of the height of the Fermi edges. It reveals the energetic position of the Highest Occupied Molecular Orbital (HOMO) respectively to the Fermi Level of the surface. Further experiments using non-monochromatized He I and He II radiation from a He discharge lamp confirm the energetic position of the HOMO maximum at 1.5–2.0 eV binding energy.

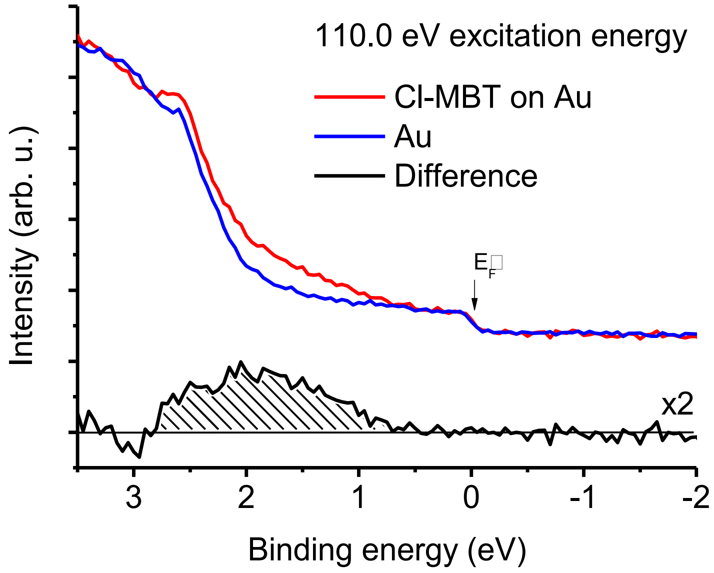

Figure S6: Valence band spectra for Au (blue) and Cl-MBT on Au taken at a photon energy of 110 eV. The difference spectrum shows the position of the HOMO of Cl-MBT; the maximum is found between 1.5 and 2.0 eV.

Resonance frequency of the tip/sample junction

The resonance of the molecule-free tunneling junction is red-shifted by about 50 nm with respect to the broad luminescence from the Au substrate.

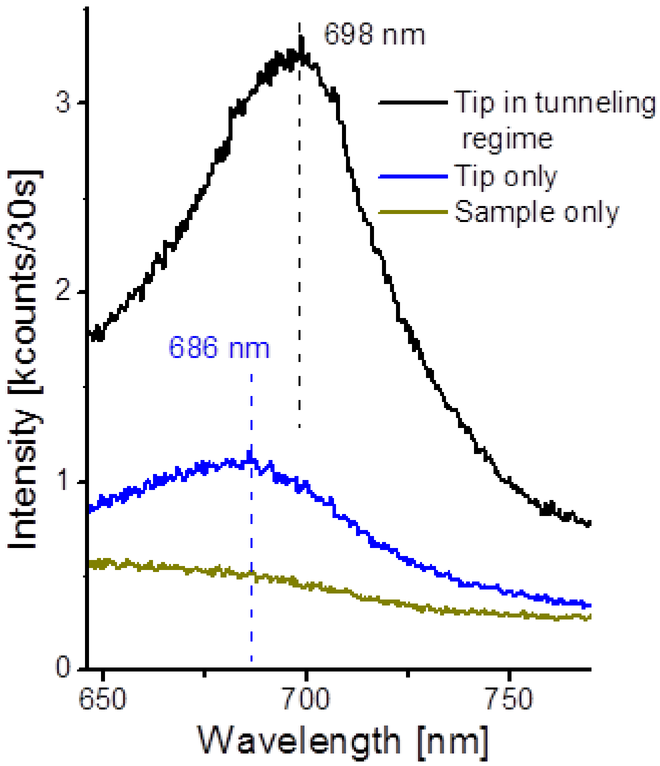

Figure S7: The graph shows the luminescence spectrum of the Au/Au-junction (black) with a resonance at 698 nm, a FWHM of 50 nm and providing a *Q*-factor of 15. Also shown is the spectrum of the freely standing tip with a maximum at 686 nm (blue), in addition to the confocal luminescence spectrum of a flat Au-substrate with a maximum around 650 nm (dark yellow). Hence the 632 nm radiation has sufficient energy to efficiently excite hot electrons which can emit photons at around 698 nm.

Tip enhanced spatial resolution

As an example to demonstrate the optical performance of our microscope we show in the following a tip enhanced Raman spectroscopic mapping of a molecular monolayer film of 2-mercapto-benzothiazole (MBT) on a smooth Au substrate. MBT binds via the sulfur atom of the end-standing thiol-group to the Au surface. Its first electronically excited state lies in UV which is far away from resonance excitation under our experimental condition. Figure S8a represents the sample topography with an area of 250 nm × 250 nm. The superimposed grid marks the location where we have recorded one TERS spectrum. With an acquisition time of 300 ms per spectrum and a total of 64 x 64 spectra we are able to record 4096 spectra in less than half an hour. The spectra shown in Figure S8c show clearly the typical Raman bands of the MBT which are excited along their long molecular axis in excellent agreement with SERS-spectra known in the literature [5]. By comparing successive spectra recorded in one row with a separation of only 3.9 nm we clearly observe differences from one to the next spectrum such as intensity variations or splitting of individual Raman peaks. This behavior is reproducible and reflects local disorder in the film and the substrate morphology. Hence we can assume that our tip enhanced optical resolution is on the order of 4 nm or better.

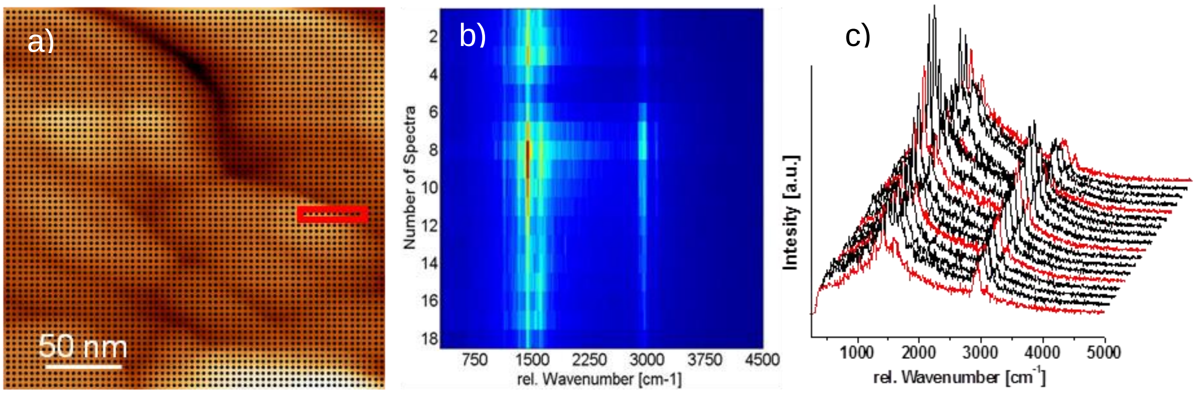

Figure S8: a) Spectral mapping of a 250 nm x 250 nm area of a monolayer film of MBT-molecules on a smooth Au substrate. Each black dot represents the position where one spectrum was collected. Excitation with λ = 632.8 nm and 250 μW. Acquisition time is 300 ms per spectrum. b) and c), taken from the red square marked area. Clear differences in successive TERS-spectra of MBT can be observed reflecting a local disorder in the molecular film and the substrate morphology.

References

1. Zerulla, D.; Uhlig, I.; Szargan, R.; Chassé, T. *Surf. Sci.* **1998**, *402–404*, 604–608.

2. Chen, J. C. *Introduction to Scanning Tunneling Microscopy*. Oxford University Press: 1993.

3. Berndt, R.; Gaisch, R.; Gimzewski, J. K.; Reihl, B.; Schlittler, R. R.; Schneider, W. D.; Tschudy, M. *Science* **1993**, *262*, 1425–1427.

4. Berndt, R.; Gimzewski, J. K.; Johansson, P. *Phys. Rev. Lett.* **1991**, *67*, 3796–3799.

5. Lee, C. J.; Lee, S. Y.; Karim, M. R.; Lee, M. S. *Spectrochim. Acta, Part A: Mol. Biomol. Spectrosc.* **2007**, *68*, 1313–1319.
